# Supplementary material for: Genetic Profiling of a Cohort of Italian Patients with ACTH-Secreting Pituitary Tumors and Characterization of a Novel USP8 Gene Variant
Source: Cancers (Basel). 2021 Aug 10;13(16):4022. doi: 10.3390/cancers13164022 (PMC8392476; doi:10.3390/cancers13164022)
Supplement: Supplementary file 1 [file cancers-13-04022-s001.zip › File S1-original western blot images.pdf]

Western blots of FLAG-USP8, pCREB, tot-CREB – Fig. 2d

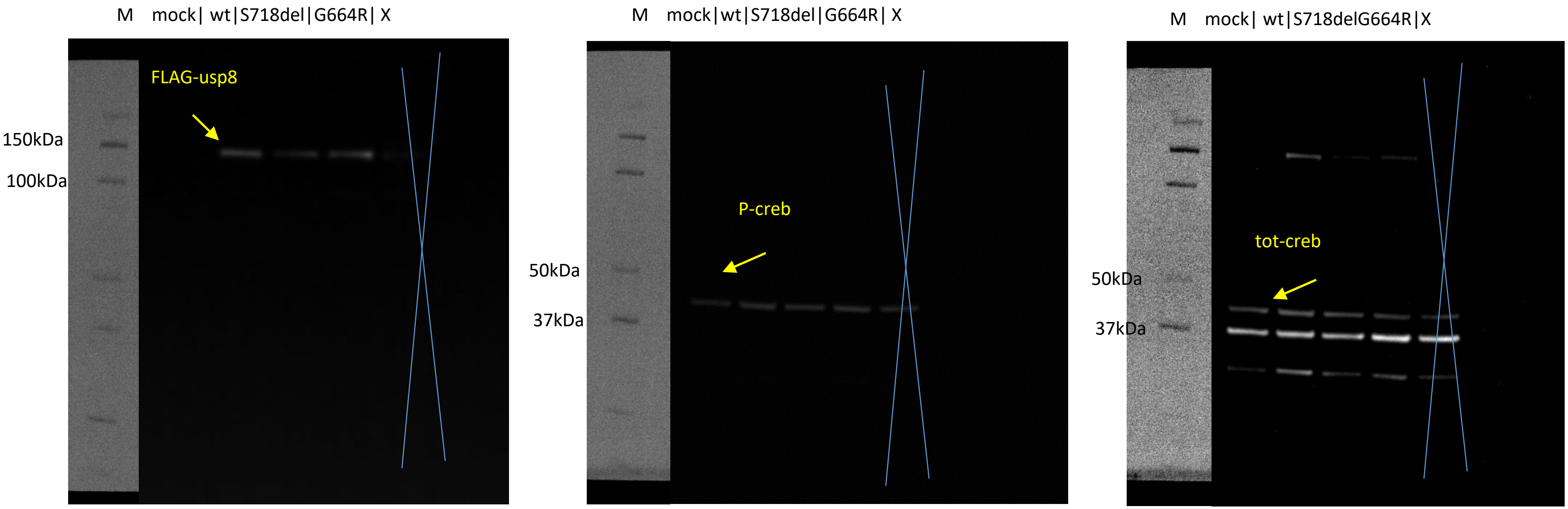

The molecular weight have been acquired in white light and juxtaposed at the original position of western blot bands

Western blots of FLAG-USP8, p27, GAPDH – Fig. 2e

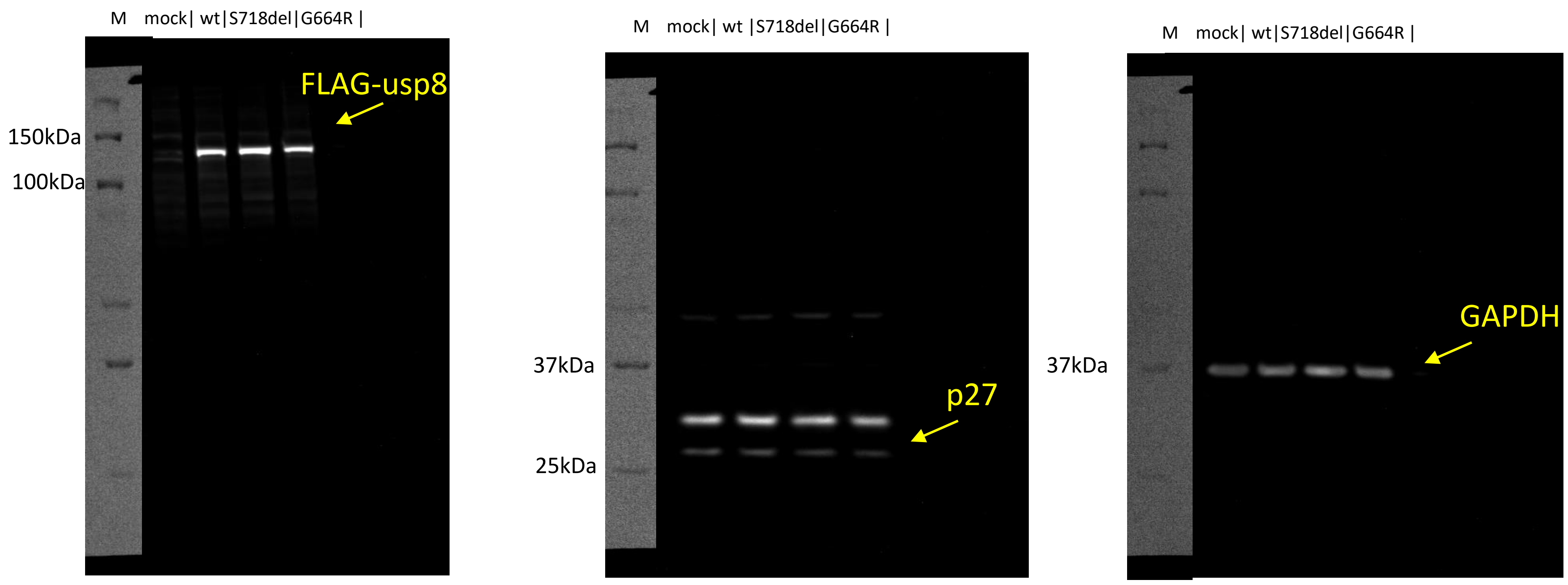

The molecular weight have been acquired in white light and juxtaposed at the original position of western blot bands

Western blots of FLAG-USP8, pERK1/2, tot-ERK1/2 – Fig.2c

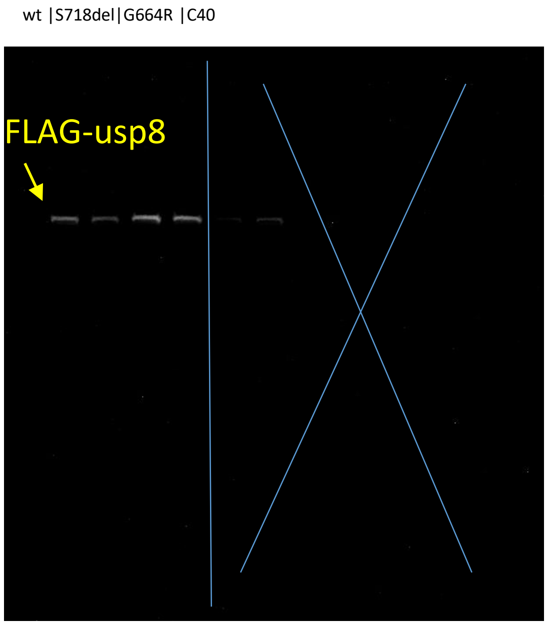

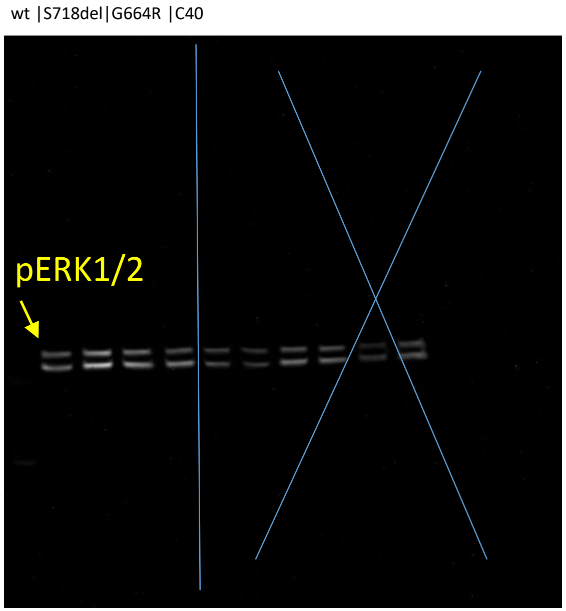

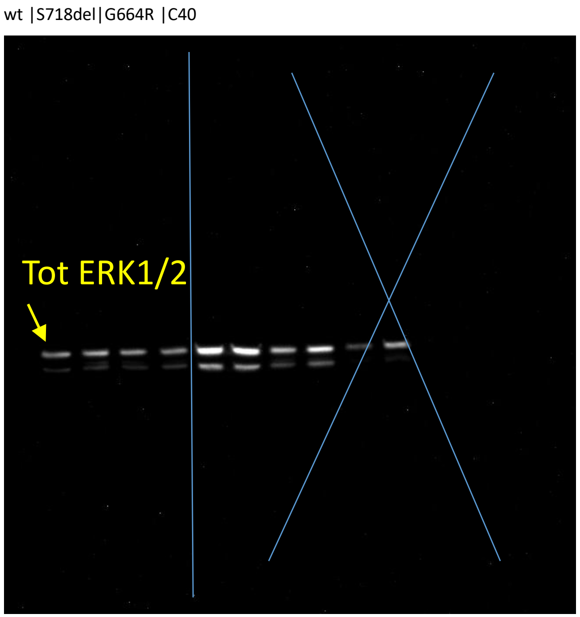

The molecular weight have not been acquired for these blots (no aspecific bands are usually detected with the above mentioned antibodies).

Western blots of FLAG-USP8, N-term USP8, GAPDH – Fig.3A

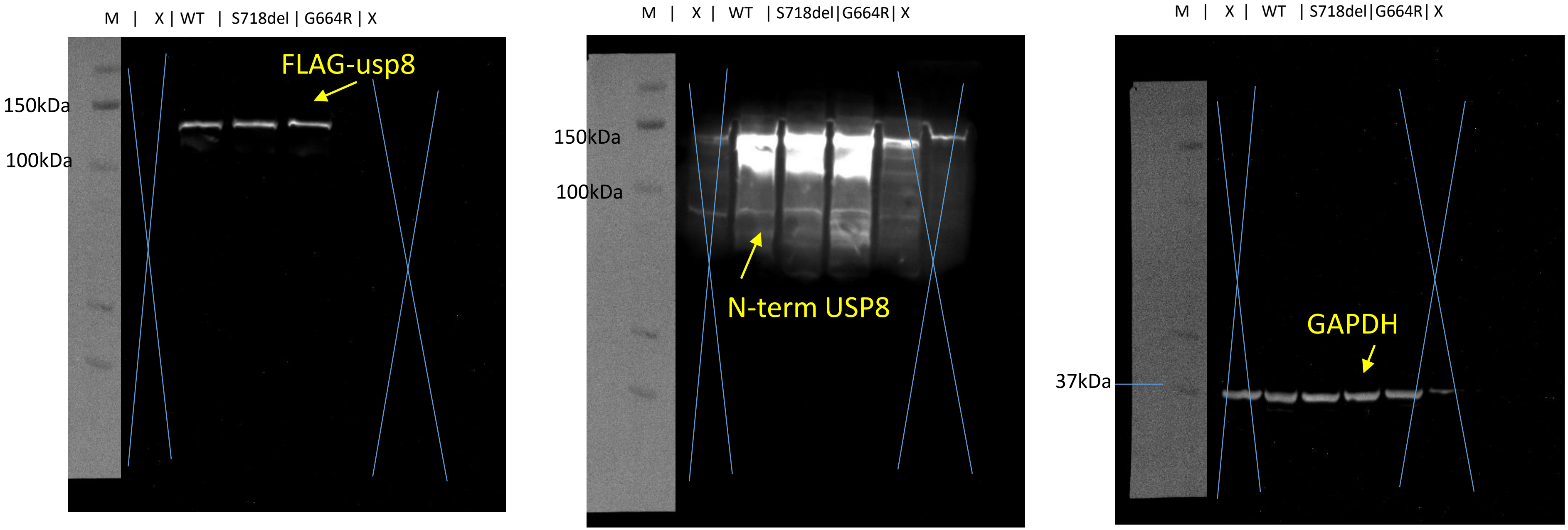

The molecular weight have been acquired in white light and juxtaposed at the original position of western blot bands
