# Supplementary material for: Genetic Profiling of a Cohort of Italian Patients with ACTH-Secreting Pituitary Tumors and Characterization of a Novel USP8 Gene Variant
Source: Cancers (Basel). 2021 Aug 10;13(16):4022. doi: 10.3390/cancers13164022 (PMC8392476; doi:10.3390/cancers13164022)
Supplement: Supplementary file 1 [file cancers-13-04022-s001.zip › supplementary tables and figures.pdf]

cancers

MDPI

Supplementary Materials

Genetic Profiling of a Cohort of Italian Patients with ACTH-Secreting Pituitary Tumors and Characterization of a Novel *USP8* Gene Variant

Donatella Treppiedi, Anna Maria Barbieri, Genesio Di Muro, Giusy Marra, Federica Mangili, Rosa Catalano, Emanuela Esposito, Emanuele Ferrante, Andreea Serban, Marco Locatelli, Andrea G. Lania, Anna Spada, Maura Arosio, Erika Peverelli and Giovanna Mantovani

Table S1. Primers used for *USP8*, *USP48* and *BRAF* hot spot amplification and Sanger sequencing.

Primer	Sequence
USP8 1F	TTTAGCAGAATACTTTGGAGTG
USP8 1R	GCTGGTATAGCCATCCACAG
USP8 2F	CTTGACCCAATCACTGGAAC
USP8 2R	TTACTGTTGGCTTCCTCTTCTC
USP8 3F	ATATGTACCCACCGGAAATG
USP8 3R	GTTCTAGGAGTTAAGATAAACATAC
USP48 1F	TCAGCAGAACCTTCTAAGTCTCA
USP48 1R	TGCCTGCTATAATCCTGGAAA
USP48 2F	GTGTTAATGTGTCTTGGGTG
USP48 2R	CACCAACATTCACCTTATGG
BRAF 1F	CATAATGCTTGCTCTGATAGGA
BRAF 1R	GGCCAAAAATTTAATCAGTGGA
BRAF 2F	GATCTCTTACCTAAACTCTTC
BRAF 2R	GTAACTCAGCAGCATCTCAG

Suppl. Fig. 1

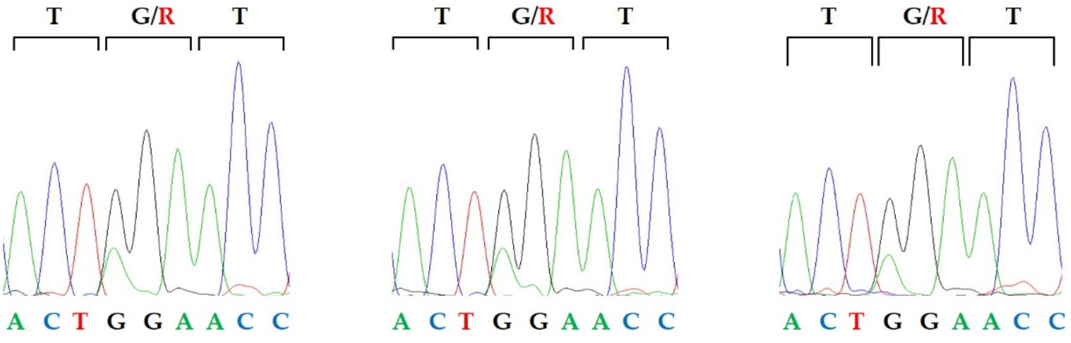

Suppl. Fig. 1. *USP8* G664R variant identification by Sanger sequencing. PCR amplification and direct sequencing were repeated three times. Encoded amino acids are shown in one-letter code; mutant amino acid is represented in red.

Figure S1: *USP8* G664R variant identification by Sanger sequencing

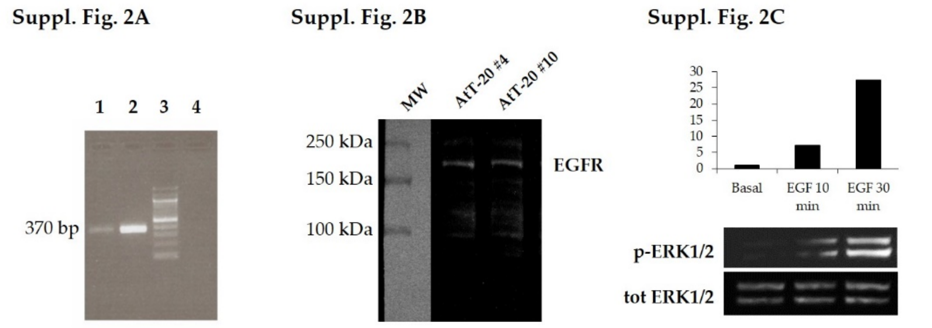

Suppl. Fig. 2. A) RT-PCR showing the presence of murine EGFR mRNA in AtT-20 cells. 1=AtT-20; 2=bone marrow (positive control); 3=100 bp marker; 4=NTC (no template control). **B) Western blot analysis of EGFR protein expression.** AtT-20 cells were tested for EGFR protein expression. 30 μg of total proteins extracted from AtT-20 cells at passages #4 and #10 were resolved by SDS page and Western blot analysis was performed with anti-EGFR antibody (Cell signalling, 1:1000 dilution). **C) EGF-mediated increase of phospho-ERK1/2.** AtT-20 cells were seeded in starved medium for 24 h, then stimulated with EGF 50 ng/ml for the indicated times and immediately chilled on ice. 30 μg of total proteins were resolved by SDS page and Western blot analysis was performed with antibodies anti phospho-ERK and anti-total ERK (Cell signalling, 1:1000 dilution).

Figure S2: RT-PCR and Western Blot analysis of EGFR expression and EGF-mediated increase of phospho-ERK1/2 in AtT-20 cells.
